# Treatment delays among women with breast cancer in a low socio-economic status region in Brazil

**DOI:** 10.1186/s12905-016-0359-6

**Published:** 2017-02-21

**Authors:** Naidhia Alves Soares Ferreira, Sionara Melo Figueiredo de Carvalho, Vitor Engrácia Valenti, Italla Maria Pinheiro Bezerra, Hermes Melo Teixeira Batista, Luiz Carlos de Abreu, Fernando Adami, Leandro Luongo de Matos

**Affiliations:** 1 Laboratory of Epidemiology and Data Analysis. School of Medicine of ABC, Santo André, SP Brazil; 2 Laboratory of Design of Studies and Scientific Writing. School of Medicine of ABC, Santo André, SP Brazil; 3 Oncológica Brasil Ensino e Pesquisa., Belém, PA Brazil; 4 Public Health Department, School of Medicine of ABC, Santo André, SP Brazil

**Keywords:** Breast neoplasms, Epidemiology, Unified health system, Hospital oncology service, Public policies

## Abstract

**Background:**

Considering the inequalities and the areas of low socioeconomic status in Brazil, access to health services is a challenge and the delay between diagnosis and treatment represents an important factor of worse prognosis in patients with breast cancer. Herein, we describe the clinical and epidemiological profiles of women with breast cancer and evaluate their access to health services, as well as treatment delays, at a reference centre of the Cariri region, Ceará, Brazil.

**Methods:**

This is a retrospective study that included 473 women treated with breast cancer between 2009 and 2011 at the Oncology Centre of the Cariri.

**Results:**

The majority of these patients were aged between 40 and 69 years old (65.7%), without a completed high school degree (89.2%). They were married (62.9%) and were already diagnosed but had not yet been subjected to any previous treatment (77.8%). It was observed that 91.8% were referred from the public health service, and treatment was paid for by the public health service in 92.9% of the cases. The patients whose source of referral was the public system waited longer between diagnosis and the treatment initiation (*p* = 0.031; Mann–Whitney’s test), with a median waiting time of 71.5 days versus 39 days for those receiving referrals from private services. In addition, those with public referrals prior to diagnosis also experienced a longer waiting time between the first medical visit and treatment initiation (77 days vs. 37 days; *p* = 0.036; Mann–Whitney’s test), with the waiting time for the biopsy being an important factor in this delay.

**Conclusions:**

Late diagnosis was often the result of inefficiency of the prevention policies coupled with difficulty accessing the public health network. It was commonly observed that, even after diagnosis, the patients needed to wait too long before entering the Oncology Service because of long waiting queues in the public health system.

## Background

The incidence of breast cancer has increased the most of all types of tumours over the last decade. Therefore, it is a major public health problem worldwide. Breast cancer, the most common malignant cancer among women, was responsible for the deaths of 522,000 women in 2012 [1]. In Brazil, approximately 57,000 new breast cancer cases were expected during 2014 [2]. In 2010, this cancer was the leading cause of death in the female population aged between 40 and 69 years old [2, 3], with 12,705 deaths [3].

Following the creation of the Unified Health System (UHS; in Portuguese called *Sistema Único de Saúde – SUS*) in Brazil, the population’s access to healthcare has increased widely. However, this health system is under development and is still working to ensure a universal and equitable coverage to fulfil its principles. Indeed, the system is highly dependent on the private sector, especially for diagnostic and therapeutic support services, in which only 28.4% of the mammography services, 51% of the ultrasound handsets, and 31.9% of the hospitals are actually public resources [4].

Because of the very large territorial dimension of Brazil, significant regional differences in the cultural, social, and economic aspects or in the occurrence of diseases exist within the country [5]. In regions of low socioeconomic status, such as southern Ceará, also known as Cariri, these differences become especially striking and ultimately generate a considerable challenge regarding the full access of the population to health services Therefore, we aimed to describe the clinical and epidemiological profiles of women with breast cancer and document their access to health services, as well as treatment delays, at a reference center in Cariri, Ceará, Brazil.

## Methods

We performed a descriptive, retrospective study of women diagnosed with breast cancer who were treated at the Oncology Centre in Cariri (OCC) between 2009 and 2011. This period was chosen due to the changes in the data acquisition system of the Cancer Hospital Registry (CHR) of the OCC after 2009, which made it the most reliable source of information. The Ethics Committee in Research of the Faculdade de Medicina do ABC approved this study (under the following number: 189.818/2013). The OCC is a reference centre for cancer treatment in the Cariri region, located south of the state of Ceará, and it receives patients from five micro-regional cities, totalling 1,405,748 inhabitants in 45 cities [6, 7], in addition to receiving patients from other states such as Pernambuco and Paraíba.

The data were obtained by analysing the CHR OCC records. The collected variables were age, education level, marital status, place of residence, year of referral to the OCC, diagnosis and previous cancer treatment, stage at diagnosis, synchronous (bilateral) tumour, laterality, family history of cancer, type of treatment received, cost of diagnosis and treatment, source of referral, date of diagnosis, date of first the appointment and date of the initiation of the treatment at the OCC. The final cohort enrolled in the study consisted of 473 women with breast cancer. Men were not included (19 exclusions).

The patients were divided into three groups according to their situation when entering the OCC:

- Group I – First consultation with prior diagnosis: this group included most of the women because they were diagnosed in the service of origin itself and not at the OCC;
- Group II – First consultation with prior diagnosis and treatment: this group corresponded to a small proportion of the patients because in these cases, the patients received diagnosis and treatment at another hospital and continued treatment at a later time at the OCC;
- Group III – First consultation without diagnosis: this group corresponded to women that were referred to the OCC with a suspected breast cancer but no formal diagnosis. Therefore, the date of the first visit to the OCC preceded the date of the diagnosis.

The delay between diagnosis and the first medical visit was calculated only for the patients in Group I because Group II patients had already been treated and Group III patients had no previous diagnosis date. All groups (Groups I, II, and III) were included in the analysis of the delay between the first medical visit and the treatment initiation. Only Groups I and III were included in the analysis of the delay between the diagnosis and treatment initiation because the patients in Group II had already been treated.

The qualitative variables were described using absolute and relative frequencies. Given the non-normality of the quantitative data using the Shapiro–Wilk test, we decided to present them based on the median, 25th percentile (p.25), and 75th (p.75) percentile, as well as their difference (interquartile range), except for the age parameter, which was considered a parametric variable. The average and standard deviation method was adopted for the descriptive data, and a Student’s *t-*test was used in the comparisons. The other statistical analyses were performed using a chi-square test or Fisher’s exact test for the qualitative variables, and the Mann-Whitney and Kruskal–Wallis tests were used for the quantitative variables. The statistical software used was Stata v11.0, and the level of significance was set at 5% (*P* < 0.05).

## Results

The full set of descriptive data is presented in Table 1. The majority of the breast cancer patients had not completed their high school education (89.2%). They were also married (62.9%) and were aged between 40 and 69 years old (65.7%). The majority were already diagnosed but had not yet been subjected to any treatment (77.8%), and had a stage I or II diagnosis (56.4%). A comparison between the early (stages I and II) and the late (stages III and IV) stage cancers revealed a homogeneity within both groups (*P* > 0.05), except in terms of the year of referral to the service. This led us to compare both groups without any selection bias and means that presentation at a late stage was not related to age, education, marital status, place of residence, or family history of breast cancer.

**Table 1:** Clinical, epidemiological, and demographic profile data and staging comparison data from a reference centre for breast cancer treatment in Cariri, Brazil, between 2009 and 2011

Variables	All groups	Stage I/II	Stage III/IV	*P*-value
Age (years) (*n* = 457)^a^	57.9 ± 14.5	57.4 ± 14.6	58.4 ± 14.2	0.492^∆^
20–29	6 (1.3%)			
30–39	51 (11.2%)			
40–49	93 (20.4%)			
50–59	109 (23.9%)			
60–69	98 (21.4%)			
70–79	71 (15.5%)			
80 or older	29 (6.4%)			
Education (*n* = 289)				
None	66 (22.8%)	30 (19.7%)	36 (24.5%)	0.195^¶^
Less than high school	192 (66.4%)	101 (66.4%)	91 (61.9%)	
High school	19 (6.6%)	13 (8.6%)	6 (4.1%)	
College	12 (4.2%)	8 (5.3%)	14 (9.5%)	
Marital status (*n* = 329)				
Single	60 (18.2%)	33 (18.2%)	27 (17.9%)	0.508^¶^
Married	207 (62.9%)	110 (61.8%)	97 (64.2%)	
Widow	50 (15.2%)	29 (16.3%)	21 (13.9%)	
Divorced	9 (2.7%)	5 (2.8%)	4 (2.6%)	
Consensual union	3 (1.0%)	1 (0.6%)	2 (1.4%)	
Place of residence (*n* = 472)				
Ceara	428 (90.7%)	250 (92.3%)	178 (88.6%)	0.730^¶^
Paraíba	4 (0.8%)	2 (0.7%)	2 (1.0%)	
Pernambuco	40 (8.5%)	19 (7.0%)	21 (10.4%)	
Year of service (*n* = 473)				
2009	132 (27.9%)	86 (31.6%)	46 (22.9%)	0.010^¶^
2010	171 (36.1%)	100 (36.8%)	71 (35.3%)	
2011	170 (36.0%)	86 (31.6%)	84 (41.8%)	
Previous diagnosis and treatment (*n* = 473)				
With diagnosis but without treatment (Group I)	368 (77.8%)	213 (78.3%)	155 (77.1%)	0.882^¶^
With diagnosis and treatment (Group II)	23 (4.9%)	12 (4.4%)	11 (5.5%)	
Without diagnosis and treatment (Group III)	82 (17.3%)	47 (17.3%)	35 (17.4%)	
Staging (*n* = 473)				
I	59 (12.2%)	–	–	–
II	213 (44.2%)			
III	169 (35.1%)			
IV	32 (6.6%)			
Synchronous tumour (*n* = 473)				
Yes	4 (0.9%)	2 (0.7%)	2 (1.0%)	0.918**
Family history of cancer (*n* = 343)				
Yes	32 (10.3%)	17 (9.9%)	15 (11.1%)	0.237^¶^
Laterality of the tumour (*n* = 321)				
Right	155 (48.1%)	87 (49.4%)	68 (46.9%)	0.284^¶^
Left	166 (51.9%)	89 (50.6%)	77 (53.1%)	

^a^Average ± Standard deviation (years); ^∆^ Student’s *t*-test; ^¶^chi-square test; **Fisher’s exact test

Concerning the characteristics of the care received by women (Table 2), we observed that most received their treatment at the OCC (90.3%), 91.8% were referred to OCC from the public health service, and the costs of diagnosis and treatment were paid by the public health service in 92.2% of the cases. Regarding the treatment modalities, as expected, chemotherapy was the first treatment for stage III and IV breast cancer patients and patients with early stage disease more frequently received hormone therapy. No other clinically relevant difference was established between the early and late stage patients.

**Table 2:** Description of the characteristics of care for women, and staging comparison from a reference centre for breast cancer treatment in Cariri, Brazil, between 2009 and 2011

Variables	All group	Stage I/II	Stage III/IV	Chi-square
Received treatment in OCC (*n* = 473)				
Yes	427 (90.3%)	245 (90.1%)	182 (90.5%)	0.864
Radiotherapy treatment in the first (*n* = 428)				
Yes	293 (68.5%)	166 (67.5%)	127 (69.8%)	0.613
Surgery in the first treatment (*n* = 428)				
Yes	212 (49.5%)	119 (48.4%)	93 (51.1%)	0.577
Chemotherapy in the first treatment (*n* = 428)				
Yes	363 (84.8%)	193 (78.5%)	170 (93.4%)	<0.0001
Hormone therapy in the first treatment (*n* = 428)^a^				
Yes	203 (47.4%)	127 (51.6%)	76 (41.8%)	0.042
Number of therapies received (*n* = 427)				
1	98 (22.9%)	61 (22.4%)	37 (20.3%)	0.728
2	107 (25.1%)	58 (21.3%)	49 (26.9%)	
3	129 (30.2%)	76 (27.9%)	53 (29.1%)	
4	93 (21.8%)	50 (28.4%)	43 (23.7%)	
Cost of diagnosis (*n* = 333)				
Public	306 (92.9%)	163 (90.6%)	143 (93.5%)	0.333
Private or health plan	27 (8.1%)	17 (9.4%)	10 (6.5%)	
Cost of treatment (*n* = 332)				
Public	306 (92.2%)	163 (91.1%)	143 (93.5%)	0.417
Private or health plan	26 (7.8%)	16 (8.9%)	10 (6.5%)	
Source of referral (*n* = 329)				
Public	302 (91.8%)	161 (89.9%)	141 (94.0%)	0.182
Private or health plan	27 (8.2%)	18 (10.1%)	9 (6.0%)	

^a^The data for oestrogen or progesterone receptor status were not available

The comparisons between the waiting times and staging, source of referral, and year of referral are detailed in the Table 3. We observed that the waiting time was similar regardless of the staging of the neoplasm. The patients referred by the public system waited longer between the diagnosis and the first medical consultation, with a median waiting time of 94 days (p.25 = 40 days and p.75 = 124 days), compared to a median of 53 days (p.25 = 15 days and p.75 = 88 days) for those referred by private services or health plans (*P* = 0.037; Mann-Whitney’s test). There was also a difference between the diagnosis and treatment initiation, with a median waiting time of 71.5 days (p.25 = 38 days and p.75 = 122.5 days), compared to a median of 39 days (p.25 = 23 days and p.75 = 89.8 days) for those referred by private services or health plans (*P* = 0.031; Mann-Whitney’s test). In particular, the patients referred in 2010 (*P* = 0.003; Kruskal–Wallis’ test) waited, on average, 78 days (p.25 = 33 days and p.75 = 99.8 days). In addition, we identified a longer waiting time between the diagnosis and first medical visit in 2010 (*P* < 0.001; Kruskal–Wallis’ test), with a median delay of 56 days (p.25 = 25 days and p.75 = 96 days). It is important to note that only the patients from Groups I and III were included in the analysis of the diagnosis-to-treatment initiation waiting time because the patients in Group II received previous treatment at a centre other than the OCC. For that reason, this period is not equal to the combination of the other two. Moreover, the time between the first medical visit and the begin of treatment, which reflects the time expending into the institution to initiate the treatment once indicated, was not different considering the period between 2009 and 2011. These data demonstrate that the treatment performed in the OCC had no change during this period of time.

**Table 3:** Comparison of the waiting time with staging, source of referral, and year of referral to a reference centre for breast cancer treatment in Cariri, Brazil, between 2009 and 2011

Variables	Waiting time (days)*		
	Diagnosis and first medical visit	First medical visit and treatment initiation	Diagnosis and treatment initiation
Groups included	I	I, II, III	I, III
Waiting time	41 (18; 83)	24 (8; 61)	69 (36.8; 119.8)
Staging			
I	58 (25.5; 96)	40.0 (24.5; 66.5)	63 (37; 142)
II	53.5 (25; 88.3)	23 (6; 59)	72 (41; 117)
III	38 (18.5; 76)	18.5 (7.3; 51.5)	62 (34; 106)
IV	22 (9; 102)	17 (4.5; 54.3)	63 (22; 127)
P**	0.440	0.497	0.802
Source of referral			
Public	94 (40; 124)	22 (7; 59.3)	71.5 (38; 122.5)
Private or health plan	53 (15; 88)	27 (7; 50)	39 (23; 89.8)
P***	0.037	0.843	0.031
Years			
2009	29 (14; 68)	26.5 (11; 74.8)	65 (40.3; 114)
2010	56 (25; 96)	22 (8; 56)	78 (47; 133)
2011	36 (15; 67)	25.5 (6.3; 60.5)	60.5 (33; 99.8)
P**	<0.001	0.297	0.003

*Median (p.25; p.75); **Kruskal–Wallis’ test; ***Mann-Whitney’s test

The subgroup analysis (Table 4) identified a longer time between the first medical visit and treatment initiation for those referred to OCC by public service without diagnosis (Group III; 77 days vs. 37 days; P = 0.036; Mann-Whitney’s test). This means that the waiting time for the biopsy was an important factor in this delay. A tendency towards a longer waiting time between diagnosis and treatment initiation in this group of patients was also noted (Group III; 47.5 days vs. 30 days; P = 0.071; Mann-Whitney’s test). Considering only the time between diagnosis and treatment initiation, it was noted that patients referred to OCC with a previous diagnosis of breast cancer had a longer waiting time in the year 2010.

**Table 4:** Subgroup analysis comparing the waiting time from first medical visit and treatment initiation and between diagnosis and treatment initiation with staging, source of referral, and year of referral to a reference centre for breast cancer treatment in Cariri, Brazil, between 2009 and 2011

Variables	Waiting time (days)*			
	First medical visit and treatment initiation	Diagnosis and treatment initiation		
Group	I	III	I	III
Waiting time	20 (43)	68.5 (84)	73.5 (89)	43.5 (58)
Staging				
I	27.5 (53)	48 (41)	95 (109)	38.5 (28)
II	20 (43)	77 (107)	78 (85)	44 (68)
III	18 (39)	74 (113)	69 (92)	54 (67)
IV	17 (45)	92 (103)	67 (99)	61.5 (92)
P**	0.594	0.564	0.659	0.721
Source of referral				
Public	19 (40)	77 (67)	74 (92)	47.5 (63)
Private or health plan	21 (55)	37 (20)	62 (97)	30 (23)
P***	0.229	0.036	0.840	0.071
Years				
2009	24 (49)	133 (149)	71 (85)	39 (68)
2010	19 (42)	77 (49)	91 (87)	49 (53)
2011	18.5 (42)	66.5 (65)	63 (89)	42.5 (64)
P**	0.138	0.510	0.012	0.478

*Median (interquartile range: difference between p.75 and p.25); **Kruskal–Wallis’ test; ***Mann-Whitney’s test

Test not performed for Group II do to few number of valid cases (*n* = 16)

## Discussion

The main finding of this study was the identification of a delay between the diagnosis and treatment initiation for the patients with breast cancer referred by the public sector of the healthcare system in northeast Brazil in Cariri, in the state Ceará. This was found to be independent of the cancer stage, especially when the biopsy had not already been performed.

Despite the efforts to increase the number of mammograms in Brazil and to detect non-palpable tumors, the mean time to diagnosis and initiation of treatment of patients with palpable tumors exceeds 180 days in most Brazilian states. The primary health care service is still deficient; there is no going guidelines for referrals and request of subsidiary exams to the health professional that attends these patients. Still, the lack of secondary references for performing the outpatient biopsy of the suspected cases, contribute to the delay of the diagnostic biopsy and long time for scheduling the specialized consultation in the referral centers [8].

The delay in further treatment is possibly a reflection of the deficit of the system in providing tertiary care services. The recognition of this deficit has motivated actions for expanding cancer care within the UHS [9]. In 2013, Law No. 12.732/2012 enforced the establishment of a maximum turnaround time of 60 days between the pathological diagnosis and the first treatment within the UHS [10]. However, there is often a delay between the diagnosis date and the first examination, as seen in this study. Considering the sequence of events, these delays may have occurred in the logging of the first visit date at the beginning of a treatment or after its diagnosis. Thus, we designed the present study to investigate the delay between the diagnosis and treatment of women of breast cancer among economically disadvantaged women in rural Brazil (Cariri, Ceará).

In another study conducted in the ABC region of São Paulo, the median waiting time was 20.5 months when calculated from the onset of the first symptoms and the logged first visit. However, the median time between the biopsy and the first treatment received was a month [11]. In Barros et al.’s [12] study covering the Federal District, 77.6% of the patients received treatment more than 90 days after the initial consultation. Trufelli et al. [13] noted that the greatest delay occurred between mammography and the adjuvant treatment, with a median of 189 waiting days.

It is considered the time for the first referenced visit to exceed 90 days as a delay in diagnosis. In Canada and England research has revealed show that the average time for specialized care and initiation of treatment is between 15 and 61 days. In Africa, East and Eastern Europe, the time to start treatment is over 7 months [8]. Eramah et al. [14] demonstrated the median for consultation in Libya was 4 months and a diagnosis of 7.5 months.

It can be inferred that the most effective strategy for the majority of the Brazilian population receiving SUS with symptomatic disease detected by the patient would be to disseminate the clinical examination of the breasts by the professionals and structure a medium-agile network for diagnostic confirmation, equipped with a mammograph, ultrasound and a team trained to perform the immediate-core biopsy, offering the anatomopathological result in seven days and immunohistochemical examination for the positive cases in Up to 30 days, ensuring the start of treatment in a short period of time [8].

In a national context, based on the analysis of the information from 139 cancer hospital records in Brazil, 75% of the patients experienced a waiting time between the diagnosis and the initiation of treatment that did not exceed three months. Between 2000 and 2008, the median waiting time ranged between 32 and 46 days. Consequently, the authors also argued that the waiting time range was shorter for the patients who were diagnosed in the hospital itself. This suggests that, possibly, the longest delay is associated with the complexity of accessing the specialised oncology units [9]. Therefore, it can be assumed that the women routed through private services follow a different path to get to the cancer treatment reference centre, which was the OCC in this study, assuring them a faster track toward treatment initiation.

The study of Barros et al. [12] corroborated the idea that the delay was associated with the incapacity of the health service itself to direct the patient through the various levels of healthcare, as established by the UHS organisation. The provision of specialist services in the UHS is in limited supply, especially within the private sector [4]. Trufelli et al. [13] also emphasised that the three most important intervals, in decreasing order of severity, are the time between mammography and the biopsy, between the biopsy and surgery, and between the result of the pathological examination and the adjuvant treatment.

In 2010, there was a longer waiting time between the diagnosis and the first consultation, as well as between the diagnosis and the initiation of the treatment. This could be explained because in the second half of 2009, the Brazilian Breast Cancer Information System (called SISMAMA in Portuguese) began to be used for the generation of computerised data related to the screening procedures, diagnostic confirmations, and follow-ups of women with abnormal examination results [15]. Therefore, financial information and reports for the procedures related to bilateral mammography, Pap smear testing and breast histology became available upon registration. The period of adaptation of the health institutions to the SISMAMA may possibly explain the longer waiting time between the diagnosis and the first consultation, as well as between the diagnosis and the initiation of treatment that occurred in 2010. In addition, this delay may be related to the administrative discontinuities and professional turnover within the management function, which was common during this period. Other factors involving structural, geographical, political, or procedural obstacles, such as the power imbalance between the members of the health network, tend to undermine the effectiveness of the government initiatives to reduce these delays [4].

Secondly, the clinical, demographical, and epidemiological profiles of the women suffering from breast cancer in that region were analysed. Similar to other published studies [12, 16, 17], the largest proportion of the studied women were aged between 40 and 69 years, which is the most common age group for the development of breast cancer [2, 18, 19].

We found that 41.7% of the patients were diagnosed at advanced stages (III and IV). This unfortunate finding is not restricted to the Northeast [2, 20], especially when analysing the population served by the public services. Gonçalves et al. [21] described this same finding in Aracaju (Sergipe) and Barros et al. [12] found that the same held true in the Federal District. However, in a domestic context, more than 75% of the tumours had already reached an advanced stage at diagnosis [2].

Although it was not statistically significant, only 33.3% of the patients referred by the private system or health plans had advanced disease, in contrast to 46.7% of those referred directly by the public system. Often, a late diagnosis is the result of the inefficiency of prevention policies and of difficulties accessing the public health network. It is also common that, even with a diagnosis in hand, the patients fail to enter at the oncology service due to the long queues of the public Brazilian Health System. This is the case mainly because much of the expert assistance within the UHS is dependent on the private sector. This ultimately generates directions that oppose the intended principle of equity and universality of the system. In this study, the diagnosis and treatment of 91.9% of the women were funded by the public system. Moreover, we could not find any relationship between age, educational level, marital status, place of residence, familial history of cancer, or the presence of synchronous or the laterality of the tumour with patients diagnosed at advanced stages, demonstrating that public education should not be restricted to some specific group of the population.

However, breast cancer is an unpleasant event that can bring traumatic experiences to the patient, such as fear of death, altered selfimage, uncertainty as to treatment and prognosis. After receiving the diagnosis, many women face personal conflicts, some have difficulty in accepting the disease, seem afraid of suffering social discrimination or even discrimination within the family, in addition to facing feelings of mutilation resulting from complete or partial removal of the breast. Thus, health education actions will be implemented in health with the family focused on the quality of life and women, a form of knowledge to improve the handles a situation [22–24].

The limitations of this study are based on the CHR not providing the specific dates of each examination, the start date of each type of treatment received, or the interval between them. For some of the variables, such as race, occupation, alcohol intake, and smoking status, no reliable data were available, mostly because they are not recorded by the professional who examines the woman in the first service (information bias). Therefore, these variables could not be analysed in this research. Another important point is the fact that Cariri reflects a poor region of Brazil. The comparisons between public and private centres of reference and the delay between diagnosis and treatment, in addition to cancer staging, could not reflect the exact reality because the great majority of the cases were refereed from the public system to the OCC.

However, these results serve as warning health authorities to implement specific policies in order to reduce the waiting time for consultation and treatment of these patients, thus leading to a better prognosis of the disease.

## Conclusions

There is an increase in the number of breast cancer cases yearly, especially among younger women, which is not specific to the areas of low socioeconomic status. A delay was identified between the diagnosis and treatment initiation, independent of the cancer stage, especially when the biopsy had not already been performed, for the patients referred by the public sector of the healthcare system in Northeast Brazil. This is an important observation that should alert health authorities to implement specific policies in order to reduce the waiting time for consultation and treatment of these patients, thus leading to a better prognosis of the disease.

## Abbreviations

CHR: Cancer hospital records
OCC: Oncology Centre in Cariri
SISMAMA: Information System Control of Breast Cancer
UHS: Unified Health System
WHO: World Health Organization
